# Digital spatial profiling of human parathyroid tumors reveals cellular and molecular alterations linked to vitamin D deficiency

**DOI:** 10.1093/pnasnexus/pgad073

**Published:** 2023-03-09

**Authors:** Chia-Ling Tu, Wenhan Chang, Julie A Sosa, James Koh

**Affiliations:** Endocrine Research Unit, Department of Medicine, San Francisco Department of Veterans Affairs Medical Center, University of California San Francisco, San Francisco, CA 94158; Endocrine Research Unit, Department of Medicine, San Francisco Department of Veterans Affairs Medical Center, University of California San Francisco, San Francisco, CA 94158; Endocrine Neoplasia Laboratory, Department of Surgery, University of California San Francisco, San Francisco, CA 94143; Endocrine Neoplasia Laboratory, Department of Surgery, University of California San Francisco, San Francisco, CA 94143

**Keywords:** parathyroid tumors, vitamin d deficiency, primary hyperparathyroidism

## Abstract

Primary hyperparathyroidism (PHPT) is a common endocrine neoplastic disorder characterized by disrupted calcium homeostasis secondary to inappropriately elevated parathyroid hormone (PTH) secretion. Low levels of serum 25-hydroxyvitamin D (25OHD) are significantly more prevalent in PHPT patients than in the general population (1–3), but the basis for this association remains unclear. We employed a spatially defined in situ whole-transcriptomics and selective proteomics profiling approach to compare gene expression patterns and cellular composition in parathyroid adenomas from vitamin D-deficient or vitamin D-replete PHPT patients. A cross-sectional panel of eucalcemic cadaveric donor parathyroid glands was examined in parallel as normal tissue controls. Here, we report that parathyroid tumors from vitamin D-deficient PHPT patients (Def-Ts) are intrinsically different from those of vitamin D-replete patients (Rep-Ts) of similar age and preoperative clinical presentation. The parathyroid oxyphil cell content is markedly higher in Def-Ts (47.8%) relative to Rep-Ts (17.8%) and normal donor glands (7.7%). Vitamin D deficiency is associated with increased expression of electron transport chain and oxidative phosphorylation pathway components. Parathyroid oxyphil cells, while morphologically distinct, are comparable to chief cells at the transcriptional level, and vitamin D deficiency affects the transcriptional profiles of both cell types in a similar manner. These data suggest that oxyphil cells are derived from chief cells and imply that their increased abundance may be induced by low vitamin D status. Gene set enrichment analysis reveals that pathways altered in Def-Ts are distinct from Rep-Ts, suggesting alternative tumor etiologies in these groups. Increased oxyphil content may thus be a morphological indicator of tumor-predisposing cellular stress.

**Significance Statement**

The pathophysiological mechanisms underlying the well-established linkage between low vitamin D status and primary hyperparathyroidism (PHPT) remain obscure. The current study employs a high-resolution cell-specific digital spatial profiling (DSP) approach to examine the downstream effects of vitamin D deficiency on parathyroid tissue. Our findings reveal that parathyroid tumors from vitamin D-deficient PHPT patients are characterized by increased oxyphil cell content and are transcriptionally distinct from tumors derived from age-matched, vitamin D-replete PHPT patients. These data suggest that vitamin D deficiency is associated with a discrete transcriptional program in parathyroid adenoma cells and imply that increased oxyphil content may arise through induced differentiation from chief cells in the context of vitamin D deficiency.

## Introduction

Low levels of serum 25-hydroxyvitamin D (25OHD) are commonly associated with primary hyperparathyroidism (PHPT) (1–3). Although parathyroid hormone (PTH) levels are known to be increased in PHPT patients with vitamin D deficiency (<20 ng/ml), the underlying pathophysiological basis for this relationship remains poorly understood (4–6). Downstream systemic effects driven by elevated PTH may enhance renal conversion of 25OHD to [1,25(OH)_2_D] with subsequent suppression of 25OHD production in the skin and liver (2, 7), but the possibility that chronic 25OHD deficiency can itself initiate changes in parathyroid tissue that predispose to adenoma development and PHPT remains an open question. Although treatment with vitamin D analogues can inhibit PTH transcription and cellular proliferation in cultured bovine parathyroid cells (8, 9), genetic ablation of the vitamin D receptor (VDR) in the parathyroid glands of transgenic mice did not induce gland hyperplasia and only modestly increased serum PTH (10), leaving the potential direct effects of 25OHD as a driver of PHPT biology largely uncertain. To investigate the potential downstream actions of 25OHD on parathyroid tissue, we performed a comparative molecular analysis of parathyroid adenomas derived from PHPT patients with preoperative vitamin D deficiency relative to tumors from vitamin D-replete PHPT patients and normal organ donor parathyroid glands.

The parathyroid glands of healthy adults are comprised predominantly of chief cells, but in conditions such as secondary hyperparathyroidism (SHPT) caused by chronic kidney disease, the relative abundance of a second parathyroid cell type known as oxyphils can increase dramatically (11, 12). Parathyroid oxyphils are characterized by high mitochondrial content and appear more abundant in older individuals (13), but the origin and physiological functions of these cells are unknown. Previously, we and others have shown that similar to chief cells, parathyroid oxyphil cells can respond to extracellular calcium stimulation via calcium sensing receptor (CaSR)-dependent signal transduction (14), express multiple parathyroid-specific factors including GCM2 and PTH (11, 15), and contain key regulatory factors such as the vitamin D-receptor, Klotho, and mitochondrial components involved in cellular respiration (16). Because oxyphil-dominant parathyroid tumors appear to drive biochemically more severe disease presentation in PHPT (17–21) and oxyphilic hyperplasia is associated with the loss of calcimimetic responsiveness in SHPT (12), we hypothesized that chronic 25OHD deficiency could produce changes in parathyroid oxyphil content and gene expression that reduce calcium sensitivity, increase PTH hypersecretion, and promote adenoma development in PHPT. To test this idea, we employed a novel, spatially indexed approach to isolate and capture oxyphil and chief cells separately from parathyroid tissue sections for subsequent transcriptomic and proteomic comparative analysis.

Here, we report that parathyroid tumors from PHPT patients presenting with preoperative vitamin D deficiency (Def-Ts) are molecularly distinct from vitamin D-replete patient tumors (Rep-Ts). The oxyphil content is markedly increased in Def-Ts, and both chief and oxyphil cells from these tumors share a discrete transcriptional signature enriched for genes involved in oxidative phosphorylation, cellular respiration, and proteasomal catabolism. In contrast, Rep-Ts are more heterogeneous and are characterized by upregulation of the *ras* and *myc* signaling pathways, suggestive of an oncogene-driven etiology. In either vitamin D context, chief and oxyphil cells are highly similar at the transcriptome level, supporting the notion that oxyphil cells are derivatives of chief cells rather than arising through an independent lineage. Interestingly, PTH transcript abundance is equivalent between normal tissue, Def-Ts, and Rep-Ts, indicating that aberrant tumor-specific PTH gene expression is not a driver of hormonal hypersecretion in PHPT. Consistent with this finding, proteomic analysis revealed that the ratio of CaSR to the type B γ-aminobutyric acid receptor (GABBR) is altered in favor of biochemically silent CaSR:GABBR heteromers (22) in Def-Ts, suggesting that CaSR antagonism could contribute to PTH hypersecretion uncoupled from calcium sensing in these tumors. Finally, a comparison between PHPT adenomas and normal parathyroid tissue independent of vitamin D status revealed that genes involved in cytoskeletal structure and tissue remodeling were significantly upregulated in the tumors, suggesting that the ability to modify cellular structure and the physical tumor microenvironment is a common denominator, acquired phenotype in PHPT adenomas. These data suggest that PHPT adenomas share certain features related to cellular structure and tissue remodeling but that vitamin D status strongly influences the intrinsic gene expression patterns and biochemical behavior of the tumors. Collectively, these results indicate that oxyphil differentiation in Def-Ts may be an indicator of metabolic stress and that vitamin D deficiency can induce gene expression changes that uncouple calcium sensing from PTH secretion.

## Results

### Parathyroid adenomas from vitamin D-deficient PHPT patients display increased oxyphil cell abundance

To determine whether the preoperative vitamin D status of PHPT patients was associated with specific changes in the cellular composition and transcriptional profile of their parathyroid adenomas, we assembled a cohort of study subjects and normal donor controls for comparative analysis (Table 1). The PHPT patients were drawn from a preexisting registry of female study subjects who underwent parathyroidectomy at our institution from 2018 to the present. Nine patients whose preoperative vitamin D levels met the Institute of Medicine definition (23) of vitamin D deficiency (25OHD ≤ 20 ng/ml) were selected, along with 34 patients who presented with a range of preoperative 25OHD levels above the vitamin D-replete threshold (25OHD ≥ 30 ng/ml). Preoperative vitamin D levels for these patients were confirmed by repeat reads of blood collected intraoperatively from each study participant. Control parathyroid glands from 12 eucalcemic organ donors were evaluated in parallel as normal reference benchmarks. The vitamin D-deficient and vitamin D-replete groups were similar in age (*P* = 0.1814), preoperative serum calcium (*P* = 0.9580), and resected gland mass (*P* = 0.9274), and all had single gland adenomas. Preoperative PTH differed significantly as has been previously reported (24), with a median of 188 pg/ml in the vitamin D-deficient group compared to 112.5 pg/ml in the vitamin D-replete group (*P* = 0.0019 by two-tailed *t*-test). The age of the normal donor controls was significantly younger than either of the PHPT cohorts (*P* = 0.001 by ANOVA).

**Table 1.** Study cohort characteristics.

Parameter	Normal donor (*n* = 12)	Vitamin D-deficient PHPT (*n* = 9)	Vitamin D-replete PHPT (*n* = 34)
Age (yr)	38 (30,74)	63 (53,71)	69 (59,74)
Sex	8 females/4 males	9 females	34 females
Pre-op. PTH (pg/ml)		188 (120.5, 240)^a^	112.5 (87.5, 141.8)^a^
Pre-op. serum Ca++ (mg/dl)		10.8 (10.45, 11.10)	10.7 (10.4, 11.1)
Gland weight (mg)		485 (264, 686)	486 (204, 695)
Pre-op. vitamin D (ng/ml)		17 (10, 18)	38 (33, 46.5)

Medians for each parameter are shown, with the 25th and 75th interquartile values in parenthesis. Gland weight refers to single gland tumors resected from patients undergoing parathyroidectomy for PHPT. Vitamin D deficiency is defined as ≤20 ng/ml, based on Institute of Medicine guidelines (2). Replete vitamin D status is defined as ≥30 ng/ml by the same guidelines.

a *P* = 0.0019 by two-tailed *t*-test for difference.

**Fig. 1.**
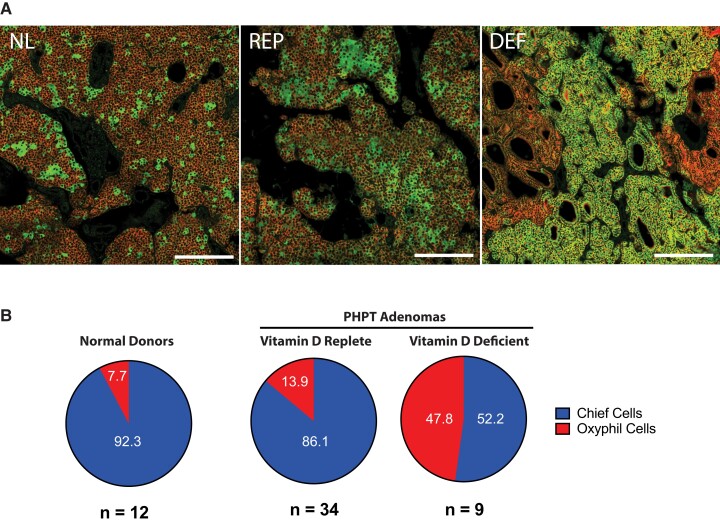
Parathyroid adenomas from PHPT patients with preoperative vitamin D deficiency contain a higher proportion of oxyphil cells. (A) The relative abundance of oxyphil cells was evaluated by immunofluorescence in tissue sections from normal donor parathyroid tissue (NL) and parathyroid adenomas from vitamin D-deficient (DEF) or vitamin D-replete (REP) PHPT patients. CaSR is stained in red to identify parathyroid cells; the mitochondrial biogenesis protein TOMM20, a marker of mitochondria-rich oxyphil cells, is stained in green. Scale bar = 0.25 mm. (B) The relative abundance of chief (blue) or oxyphil cells (red) were quantitated in a panel of normal (*n* = 12) or adenoma tissue sections from vitamin D-deficient (*n* = 9) or vitamin D-replete (*n* = 34) PHPT patients. Percentages are based upon cell counts from full-section low-power fields for each tumor.

To investigate the relative abundance of chief and oxyphil cells in the adenomas from each vitamin D group, formalin-fixed, paraffin-embedded sections were prepared and examined by immunofluorescence. To exclude vascular components, adipocytes, and other nonparathyroid cell types, the sections were stained for the calcium sensing receptor (CaSR), since elevated abundance of this protein is a recognized hallmark of parathyroid cells. The sections were costained for TOMM20 (*translocase of outer mitochondrial membrane 20*), a mitochondrial marker that is highly enriched in parathyroid oxyphil cells. To maintain consistent staining conditions for comparative purposes, each glass slide included tissue sections from a normal parathyroid gland (Fig. 1A, NL), an adenoma from a vitamin D-replete patient (Fig. 1A, REP), and an adenoma from a vitamin D-deficient patient (Fig. 1A, DEF). The entire specimen area of each slide was scanned with a 20× objective in a GeoMx Digital Spatial Profiler (DSP). The proportions of CaSR-positive cells expressing high (oxyphil cells) versus low (chief cells) levels of the TOMM20 marker were then quantified by ImageJ and expressed as a fraction of the total cell number determined by SYTO13-positive nuclear counts of the CaSR-positive population.

As expected, normal parathyroid tissue contained the lowest proportion of oxyphil cells (7.7%), likely due in part to the younger age of the donor group, as the oxyphil content is known to increase with age (25). In the age-matched PHPT patient cohorts, Def-Ts had significantly greater oxyphil abundance (Fig. 1B), accounting for nearly half (47.8%) of the cellular content of the tumors on average. In contrast, oxyphil abundance was much lower in Rep-Ts (13.9%). The oxyphil cells in Def-Ts tended to occur in large, contiguous areas as opposed to the more scattered pockets or isolated cells observed in normal tissue or in Rep-Ts.

### Transcriptional spatial profiling reveals distinct gene signatures in parathyroid adenomas from PHPT patients with preoperative vitamin D deficiency

**Fig. 2.**
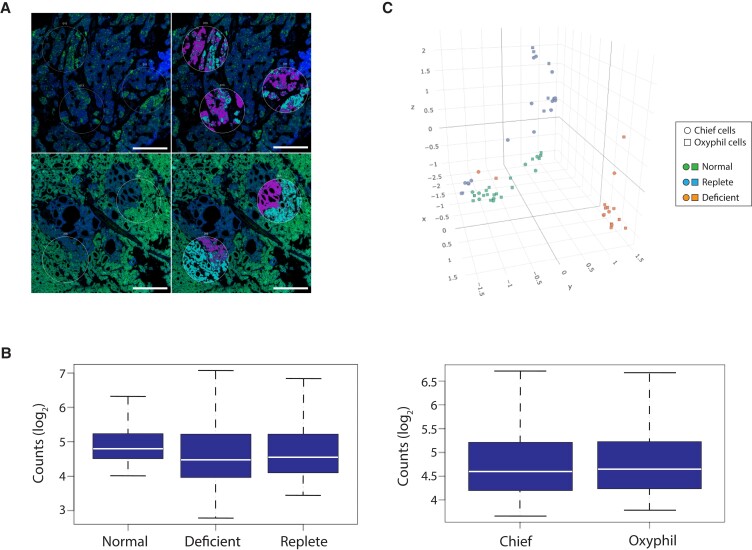
Transcriptional spatial profiling reveals discrete gene signatures in parathyroid adenomas from PHPT patients with preoperative vitamin D deficiency. (A) Tissue sections were stained with Syto13 (blue) to visualize nuclei and anti-TOMM20 (green) to identify oxyphil cells. The upper panels are from a tumor from a vitamin D-replete patient. The lower panels are from a vitamin D-deficient patient. Regions of interests (ROIs) were selected (white circles) that included both chief (TOMM2−) and oxyphil (TOMM20+) cells. Chief and oxyphil cells were captured separately from each other in each ROI using the indicated masks (teal = oxyphil cells; purple = chief cells). Scale bar = 0.25 mm. (B) Distribution of total Q3 normalized, log2 transformed counts per gene by the tissue group (left) or by cell type (right). Upper and lower box boundaries represent the 75th and 25th percentiles of each data group. The white horizontal line indicates the median, and error bars indicate the standard deviation. (C) Principal component analysis visualized in a three-dimensional tSNE plot. Green symbols = normal parathyroid tissue. Blue symbols = tumors from vitamin D-replete patients. Orange symbols = tumors from vitamin D-deficient patients. Circles = chief cells. Squares = oxyphil cells.

We then sought to determine whether the gene expression profiles of Def-Ts and Rep-Ts were similar or distinct. Because the oxyphil content of these tumor groups was clearly different, we employed a digital spatial profiling (DSP) approach to capture chief and oxyphil cells separately for direct transcriptional comparisons between cell types from each tissue specimen. Multiple regions of interest (ROIs) encompassing both cell types were selected from each formalin-fixed, paraffin-embedded tissue section, and CaSR+/TOMM20-high (oxyphil cells) and CaSR+/TOMM20-low (chief cells) were marked for selective capture (Fig. 2A). A GeoMx DSP instrument (NanoString Technologies) was used to interrogate gene expression within the chief or oxyphil cells separately captured from each ROI.

Utilizing a human whole transcriptome oligonucleotide library and next-generation sequencing, we performed a quantitative analysis of over 18,000 unique transcripts in the targeted cells captured from each ROI. Quality control metrics including target gene saturation, total transcript counts, and mean counts per transcript were evaluated for each tissue source (normal, Def-Ts, or Rep-Ts) and for each cell type (chief vs oxyphil) (Table S1, Fig. S1). No significant differences in these metrics were observed between input groups. For example, the mean counts per transcript did not vary when comparing either tissue source or cell type (Fig. 2B).

The raw sequencing counts were normalized through the Q3 (third quartile of all targets above the limit of quantitation) method (26), using the top 25% of expressors to normalize across all ROIs and captured cell subsets. The normalized data were then subjected to principal component analysis to visualize gene expression effects associated with the cell type or vitamin D status. In a three-dimensional *t*-distributed stochastic neighbor embedding (tSNE) plot, both chief and oxyphil cells from Def-Ts formed a discrete cluster widely separated from normal parathyroid tissue and from Rep-Ts (Fig. 2C). Two-dimensional projections from UMAP and PCA modeling generated similar results (Fig. S2). Compared to cells from Def-Ts, chief and oxyphil cells from Rep-Ts are grouped more loosely and, in some cases, appeared to be closely related to normal tissue. Notably, both chief and oxyphil cells segregated by tissue source rather than by cell type. In one specific case, the chief cell and oxyphil cell inputs from a single Def-T appeared as outliers grouping more closely to vitamin D-replete tumor tissue; this patient's preoperative vitamin D level was the highest in the deficient group (25OHD = 18.2 ng/ml).

### Unsupervised hierarchical cluster analysis identifies gene signatures associated with vitamin D status in parathyroid tumors

**Fig. 3.**
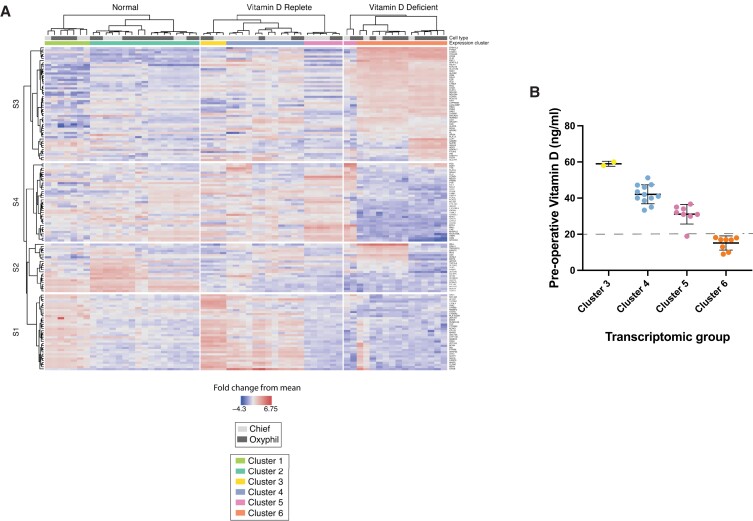
Digital spatial profiling reveals distinct transcriptional pathway changes in parathyroid tumors from vitamin D-replete and vitamin D-deficient PHPT patients. (A) Q3 normalized counts were compared across normal parathyroid tissue and parathyroid adenomas from vitamin D-deficient or vitamin D-replete PHPT patients and analyzed by unsupervised two-way hierarchical clustering. The DSP heatmap shows the top 150 differentially expressed genes with signature pattern relationships shown on the left of the heatmap. (B) Preoperative vitamin D levels show dosage effects (*P* < 0.0001 by ANOVA) correlating with gene expression clusters of PHPT patients. Serum 25OHD mean and standard deviations for each cluster group are shown, with each dot representing an individual patient value. The dotted line demarcates 20 ng/ml, the established threshold for vitamin D deficiency.

To visualize transcriptome profiles potentially associated with vitamin D status, the 12,762 unique transcripts detected were rank ordered by the degree of differential expression, and unsupervised hierarchical cluster analysis was performed using the ComplexHeatmap R/Bioconductor package. Genes encoded on the X and Y chromosomes were excluded to remove sex as a differentiating variable, as four of the normal tissue donors were male. Consistent with the PCA results, the interrogated samples segregated by vitamin D status, with normal parathyroid tissue clustering separately from both tumor groups (Fig. 3A). The cell type did not emerge as a primary organizing variable, as chief cells and oxyphil cells both clustered within each tissue group rather than segregating independently. A total of six transcriptional profile clusters emerged: two from normal parathyroid tissue (clusters 1 and 2), three within Rep-Ts (clusters 3, 4, and 5), and two (clusters 5 and 6) within the Def-Ts. The chief and oxyphil cells from the one outlier vitamin D-deficient patient sample noted in the PCA plot sorted into cluster 5, a profile shared with eight Rep-T specimens. Cluster 6 represented the predominant signature of the Def-Ts, encompassing tumors from eight of the nine vitamin D-deficient patients. The two clusters from normal tissue each contained both chief and oxyphil cells. The tumors from the Rep-Ts were the most heterogeneous, with three different profile clusters identified. Strikingly, the three clusters visualized among Rep-Ts correlated with the mean preoperative vitamin D levels of the patients in each group (Fig. 3B), demonstrating a potential dose-dependent relationship between tumor transcriptional profile patterns and vitamin D status. The differences in the mean preoperative 25OHD levels of the patients in each Rep-T transcriptional cluster were highly significant (*P* < 0.0001 by ANOVA). Cluster 6, the Def-T group which by definition had the lowest mean 25OHD level, segregates as a distinct transcriptional profile and does not appear to be closely related to any of the three Rep-T profiles.

Pathway analysis of the DSP data revealed that genes associated with oxidative phosphorylation, mitochondrial electron transport chain function, and the citrate TCA cycle were significantly upregulated in both chief and oxyphil cells from the Def-Ts relative to normal tissue. Surprisingly, oxyphil cells from normal tissue and from Rep-Ts did not display upregulation of these same pathways despite the high mitochondrial content of these cells. Relative to Def-Ts, Rep-Ts were more heterogeneous and demonstrated a greater degree of overlap with normal tissue expression patterns. While vitamin D deficiency is associated with a distinct pattern of gene expression, the correlation between Rep-T clusters and preoperative vitamin D levels in the absence of a readily apparent unifying profile suggests that additional variables beyond vitamin D status contribute to transcriptomic heterogeneity in PHPT adenomas.

To evaluate potential functional differences between the groups, gene set enrichment analysis was performed (27). Annotated databases from the curated Gene Ontology/Biological Processes (GOBP), Kyoto Encyclopedia of Genes and Genomes (KEGG), and GSEA Molecular Signatures Database (MSigDB) repositories were queried for association with the transcriptome profiles revealed in the hierarchical cluster analysis. Four phenotype-associated signatures of differentially expressed genes were identified (Table 2). Rep-Ts were preferentially associated with activation of Myc target genes (MSig DB: M5926, correlation coefficient 0.811), genes regulated in response to amyloid beta (GOBP: 1904645, correlation coefficient 0.747), and genes activated by K-ras signaling (MSigDB: M5953, correlation coefficient 0.632). In contrast, Def-Ts were strongly associated with genes involved in oxidative phosphorylation (MSigDB: M5936, correlation coefficient 0.942) and to a lesser extent genes linked to proteasomal catabolism (GOBP: 0010499, correlation coefficient 0.587) and the TCA cycle (KEGG: M3985, correlation coefficient 0.546). Genes differentially expressed in normal parathyroid tissue were strongly associated with downregulation of K-ras signaling (MSigDB: M5956, correlation coefficient 0.942) and regulation of calcium transport (GOBP: 0051924, correlation coefficient 0.545). A fourth signature shared by both normal tissue and Rep-Ts was most closely associated with genes involved in fat-soluble vitamin metabolic processes (GOBP:0006775, correlation coefficient 0.570).

**Table 2.** Gene set enrichment analysis.

Signature	Sample group	Gene set	Gene set ID	Correlation coefficient
S1	25OHD-replete PHPT	Myc targets	MSigDB: M5926	0.811
		Response to amyloid beta	GOBP: 1904645	0.747
		K-ras signaling UP	MSigDB: M5953	0.632
S2	Normal parathyroid	K-ras signaling DOWN	MSigDB: M5956	0.942
		Regulation of Ca++ transport	GOBP:0051924	0.545
S3	25OHD-deficient PHPT	Oxidative phosphorylation	MSigDB:M5936	0.982
		Proteasomal catabolism	GOBP: 0010499	0.587
		TCA cycle	KEGG:M3985	0.546
S4	Normal, replete	Fat soluble vitamin metabolic processes	GOBP: 0006775	0.570

Gene set enrichment analysis was performed using unsupervised cluster data. Gene sets with the highest correlation coefficients for association with each sample group are listed. GOBP, Gene Ontology Biological Processes database; MSigDB, Broad Institute Gene set Enrichment Analysis Molecular Signatures Database; KEGG, Kyoto Encyclopedia of Genes and Genomes.

### Chief and oxyphil cells share highly similar transcriptional profiles, while vitamin D status is associated with gene expression changes that affect both cell types

**Fig. 4.**
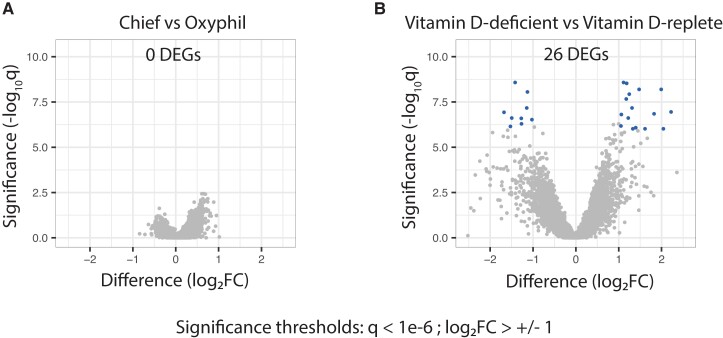
(A) Chief and oxyphil cells share highly similar gene expression patterns; (B) vitamin D status is associated with gene expression changes that affect both cell types. Volcano plots depict the log_2_(fold change) in individual gene expression between the indicated comparison groups on the *x*-axis and log_10_(adjusted statistical significance) on the *y*-axis. Genes highlighted in blue had a log_2_(fold change) value of greater than 1 and a false discovery rate (FDR) significance threshold of <1e^−06^.

To identify individual genes with the greatest degree of differential expression between cell types and vitamin D status groups, we performed a series of pairwise comparisons employing the DESeq Wald test, edgeR quasi-likelihood *F* test, and limma.zoom, each implemented as Bioconductor modules in R (28–30). When comparing chief cells to oxyphils, either collectively or considering the two cell types in each vitamin D status group separately, no genes met a differentially expressed gene (DEG) threshold of log2(fold change) > ±1 and *q* < 1e^−6^ (Fig. 4A). In contrast, when comparing Def-Ts to Rep-Ts, 26 DEGs were detected that met the same differential expression criteria (Fig. 4B). The DEGs, their fold change in the Def-Ts relative to the Rep-Ts, and the corresponding *q*-values are listed in Table 3. EPB41L3 (*erythrocyte membrane protein band 4.1-like 3*), a cytoskeleton protein–membrane anchor with suspected tumor suppressor properties (31, 32), showed the greatest fold change, with a mean fold increase of almost 16-fold in Def-Ts. This gene has been found to be upregulated in benign meningiomas, while its loss by mutational inactivation or gene silencing has been associated with enhanced invasiveness and malignant transformation in gastric and colorectal cancers (32). Expression of MAPK8IP1 (*mitogen-activated protein kinase 8 interacting protein 1*), a key regulatory protein that opposes MAPK8-mediated activation of downstream transcription factors and colocalizes with amyloid deposits in the neurofibrillary tangles of Alzheimer's disease patients (33), is significantly diminished in Def-Ts. Notably, expression of GABBR1 (*gamma aminobutyric acid type B receptor 1*), which our group has recently shown to oppose CaSR-mediated calcium sensing in the parathyroid (34), is significantly elevated in Def-Ts. Additional genes found to be significantly upregulated in these tumors act in signaling pathways controlling apoptosis (TNFSF10, *tumor necrosis superfamily member 10*) (35, 36), cellular stress response (SGK1, *serum glucocorticoid regulated kinase 1*) (37), inositol-derived second messenger production (IPMK, *inositol polyphosphate multikinase*) (38), and energetic metabolism (AMPD3, *adenosine monophosphate deaminase 3*) (39). Despite the higher preoperative PTH observed in vitamin D-deficient PHPT patients, PTH transcript abundance was not significantly different between tissue sources (*P* = 0.2862 by one-way ANOVA) or when comparing chief to oxyphil cells (*P* = 0.7284 by unpaired *t*-test). These data suggest that alterations in secretory or sensing mechanisms, rather than aberrant overexpression of PTH transcription, are the primary drivers of PTH hypersecretion in PHPT.

**Table 3.** Genes differentially expressed between tumors from vitamin D-deficient and vitamin D-replete PHPT patients.

Gene	Log2(fold change)	*q*
EPB41L3	3.934	5.39 E^−19^
MAPK8IP1	−1.417	2.64 E^−09^
GABBR1	1.112	2.64 E^−09^
TNFSF10	1.185	2.95 E^−09^
IPMK	1.475	6.35 E^−09^
SGK1	1.990	6.35 E^−09^
AMPD3	1.130	8.79 E^−09^
PPARGC1A	1.245	1.17 E^−08^
MAL2	1.175	2.16 E^−08^
SERINC2	1.310	6.74 E^−08^
YIF1A	−1.146	6.74 E^−08^
COL6A6	2.220	1.11 E^−07^
SEZ6L2	−1.676	1.17 E^−07^
KCNJ13	1.824	1.42 E^−07^
GLS	1.061	1.55 E^−07^
SFT2D1	1.222	2.42 E^−07^
MELTF	−1.496	2.43 E^−07^
NAV2	−1.273	2.53 E^−07^
LARGE2	−1.025	2.98 E^−07^
SYT11	−1.269	5.07 E^−07^
RGS9	1.052	6.68 E^−07^
COL13A1	−1.527	7.06 E^−07^
AIF1L	1.397	8.27 E^−07^
PLAT	2.041	9.54 E^−07^
AFAP1L2	2.041	9.55 E^−07^
S100A14	1.613	9.56 E^−07^

DEGs are listed in ascending order of *q*-value (*P*-value for significance adjusted for FDR). Log_2_(fold change) represents the mean relative difference in expression between tumors from vitamin D-deficient PHPT patients compared to tumors from vitamin D-replete patients. Positive log_2_(fold change) values indicate higher expression in tumors from vitamin D-deficient patients.

### Increased GABBR protein abundance in tumors from vitamin D-deficient PHPT patients favors the formation of biochemically inactive CaSR/GABBR heteromers

**Fig. 5.**
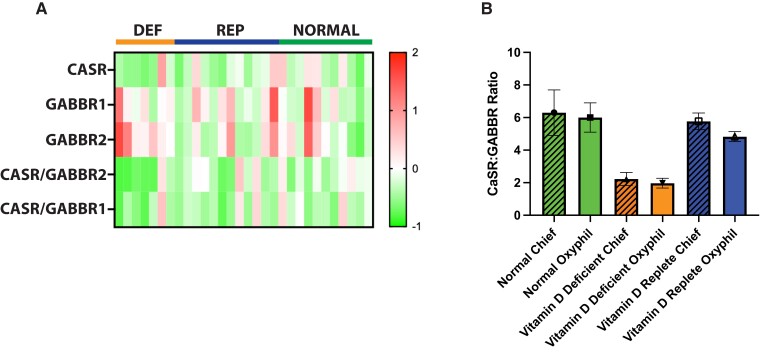
Inactive CASR/GABBR heteromer formation is favored in tumors from vitamin D-deficient PHPT patients. (A) Protein abundance of CASR, GABBR1, and GABBR2 was determined in situ from parathyroid tissue sections using a NanoString DSP instrument and a custom conjugated antibody panel. The relative abundance of the individual proteins and the ratio of CASR to the GABBA receptor proteins were determined in sections from vitamin D-deficient patient tumors (DEF), vitamin D-replate patient tumors (REP), or normal donor parathyroid tissue (NORMAL). (B) The ratio of CaSR protein to GABBR proteins was determined in the indicated cell types and tissue sources. Green bars = normal tissue. Orange bars = tumors from vitamin D-deficient patients. Blue bars = tumors from vitamin D-replete patients. Hashmarked bars = chief cells. Plain bars = oxyphil cells. Values shown are the means ± standard deviation for each sample group. *n* = 6 (normals), *n* = 13 (replete), and *n* = 6 (deficient).

The modest but statistically significant increase in GABBR1 transcript abundance in Def-Ts suggested that the stoichiometric balance between active, calcium-responsive CaSR:CaSR homomers relative to signaling-attenuated CaSR:GABBR heteromers (22) might be shifted in favor of the inactive complex in Def-Ts. To assess this notion, we utilized the DSP platform to determine the protein abundance of CaSR, GABBR1, and GABBR2 in normal parathyroid tissue sections and in Def-Ts or Rep-Ts. The protein abundance of both GABBR variants was higher in Def-Ts, while CaSR abundance in these tumors was consistently lower (Fig. 5A). These changes resulted in a significantly reduced ratio of CaSR to GABBR proteins in both chief and oxyphil cells from vitamin D-deficient patients, while Rep-Ts were indistinguishable from normal tissue in this assay (Fig. 5B).

### Parathyroid adenomas as a group express higher levels of genes involved in tissue remodeling and cytoskeletal function

**Fig. 6.**
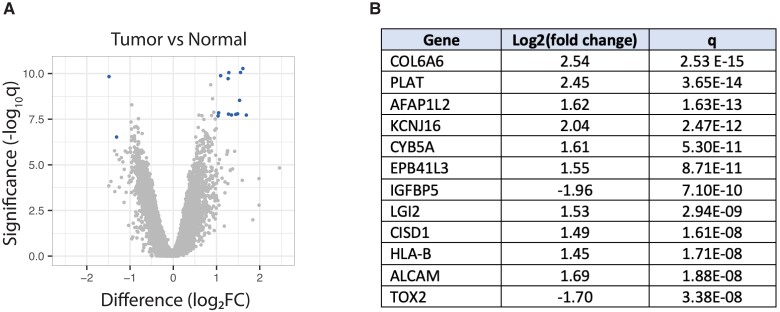
Genes differentially expressed between parathyroid adenomas and normal parathyroid tissue. (A) Volcano plot of differentially expressed genes, depicted as described in Fig. 4. (B) Differentially expressed genes listed in descending order by *q*-value, with the log2(fold change) values representing expression in tumor tissue relative to normal tissue.

When we compared all the parathyroid adenomas in our cohort as a group to normal parathyroid tissue using the same DEG criteria described above, twelve genes emerged as differentially expressed (Fig. 6A). Ten genes were upregulated in the tumors relative to normal tissue, while two genes were downregulated relative to normal tissue (Fig. 6B). The top three (ranked by *q*-value) and six of the 10 upregulated genes have roles in cellular structure and tissue architecture. COL6A6 (*collagen type VI alpha 6 chain*) is a component of the basal lamina of epithelial cells and plays a central role in maintaining extracellular matrix structure and function (40). PLAT (*tissue-type plasminogen activator*) is a secreted serine protease whose enzymatic action is essential for cell migration and tissue remodeling (41). AFAP1L2 (*actin filament-associated protein 1-like 2*) is an adaptor protein whose elevated expression is associated with the epithelial–mesenchymal transition, cellular migration, and tissue repair (42–44). EPB41L3, cited earlier, is a cytoskeletal protein anchor, and ALCAM (*activated leukocyte cell adhesion molecule*) has been shown to play an important role in invasive cellular behavior, mesenchymal stromal cell activity, and extracellular vesicular trafficking (45–47). TOX2 (*TOX high-mobility group box family 2*) is a transcriptional coactivator that modulates multiple pathways including tissue remodeling and tumor microenvironment functions (48). One of the two downregulated genes, IGFBP5 (*insulin-like growth factor binding protein 5*), is a key regulator of osteogenic differentiation, and agents that antagonize its expression have been shown to promote osteoporosis (49, 50).

## Discussion

The current paradigm for explaining the well-established association between low vitamin D levels and PHPT posits that 25OHD hypovitaminosis is a consequence of constitutively elevated PTH. Multiple mechanisms to support this idea have been proposed, including PTH-mediated suppression of 25OHD synthesis, shortened serum half-life, and restricted bioavailability (2, 51–53). Here, we have explored an alternative viewpoint by investigating whether vitamin D deficiency can exert downstream effects on parathyroid tissue, testing the hypothesis that low vitamin D status could act as a potential driver of PHPT development and PTH hypersecretion. Our data reveal that parathyroid adenomas from PHPT patients with preoperative vitamin D deficiency (Def-Ts) are intrinsically different from tumors from vitamin D-replete PHPT patients (Rep-Ts) with respect to cellular content and transcriptional profile. The cellular composition of Def-Ts reflects a striking increase in the relative abundance of parathyroid oxyphil cells compared to age-matched Rep-Ts. At the transcriptional level, genes involved in cellular respiration are preferentially upregulated in Def-Ts, while Rep-Ts are more heterogeneous, showing enhanced expression of genes in ras and myc oncogene-activated pathways and beta-amyloid protein signaling. These differences suggest that the respective etiologies of Def-Ts and Rep-Ts may be distinct, with the former being driven by adaptive metabolic responses and the latter by oncogenic signal transduction pathway activation.

The increased oxyphilic content of Def-Ts is reminiscent of reports characterizing the hyperplastic parathyroid glands of patients with chronic kidney disease who have developed secondary hyperparathyroidism (SHPT) (11–13, 16). In the most recent of these studies, Mao and coworkers compared chief and oxyphil cell nodules from the parathyroid glands of uremic SHPT patients and found that the oxyphil cells were enriched for mitochondrial proteins; expressed lower levels of proliferation-associated genes and regulatory factors such as the vitamin D receptor, Klotho, and CaSR; and secreted higher levels of PTH (16). This cellular phenotype is similar to Def-Ts, where mitochondrial genes are upregulated in the absence of a proliferative signature, and higher PTH secretion is observed relative to Rep-T patients. However, several important distinctions between the findings of the Mao study and the data that we report here suggest that calcimimetic-resistant SHPT and vitamin D deficiency-associated PHPT arise through different underlying molecular mechanisms.

In Def-Ts, both oxyphil and chief cells display upregulation of oxidative phosphorylation, electron transport chain, and TCA cycle components, suggesting that both cell types have mobilized a response to increased energetic demand. This signature is not apparent in oxyphils from Rep-Ts or normal parathyroid tissue, indicating that the increased mitochondrial content of oxyphil cells alone does not explain the higher transcript levels of the cellular respiration genes. The absence of a purely mitochondrial signature in all oxyphils regardless of tissue source argues against compensatory mitochondrial biogenesis caused by mitochondrial mutations (25) in our vitamin D-deficient cohort. In contrast to the divergent chief vs oxyphil signatures observed in the hyperplastic glands of SHPT patients (16), the high degree of transcriptional similarity between chief and oxyphil cells within individual parathyroid glands in our study suggests that vitamin D deficiency exerts the same effect on both cell types in parathyroid adenomas. The fact that oxyphil and chief cells share expression of definitive parathyroid markers and retain highly similar transcriptomic profiles that respond similarly to vitamin D deficiency is consistent with oxyphils being a derivative of chief cells. Because the greater oxyphil content of Def-Ts does not appear to be accompanied by increased cellular proliferation relative to Rep-Ts, it is most likely that these cells arise through postmitotic differentiation of preexisting chief cells rather than through expansion of an independent cellular lineage. The increased oxyphil content in Def-Ts could indicate that this phenotypic differentiation is induced in response to vitamin D deficiency.

Parathyroid adenomas are benign, relatively indolent neoplastic lesions characterized by a low mitotic index (54, 55), but activating mutations in proliferation-inducing oncogenes such as cyclin D1 have been shown to occur in 20–40% of sporadic PHPT tumors (56). Our data are consistent with Rep-Ts arising through growth-promoting mechanisms, as genes associated with increased ras signaling and myc activation are preferentially expressed in these tumors but not in Def-Ts. Conversely, genes associated with downregulation of ras signaling are enriched in normal tissue, suggesting that deregulation of this pathway is a key event in the etiology of Rep-Ts. Supporting this idea, cyclin D1 transcription is modestly elevated in Rep-Ts compared to Def-Ts (log2(fold change) = 0.325; *P* = 0.045). Rep-Ts and normal parathyroid tissue retain the expression of genes involved in fat-soluble vitamin metabolic processes, while Def-Ts lose this signature, consistent with the concept that Rep-Ts may be driven more by proliferative changes than by metabolic disruption. Rep-Ts also retain sensitivity to vitamin D, displaying three distinct transcriptional profile clusters that correlate with preoperative vitamin D levels. This heterogeneity suggests that Rep-Ts preserve the capacity to respond to vitamin D levels and implies that the etiology of these tumors is not dependent upon the absence or diminution of 25OHD-mediated signaling.

In contrast to the oncogene-activated signature of Rep-Ts, Def-Ts are characterized by differential expression of genes involved in pathways linked to opposition of cellular invasiveness, MAPK signaling, calcium sensing, and cellular stress response. EPB41L3, a protein–membrane anchor, is highly overexpressed in Def-Ts and has been found to exhibit tumor suppressor properties in multiple other tissues, with increased expression in early-stage benign tumors and loss or inactivation upon malignant transformation and the onset of invasive cellular behavior (57). It is possible that EPB41L3 upregulation in the Def-T subset of parathyroid adenomas reflects a self-limiting protective mechanism similar to that seen with other tumor suppressor gene pathways in early-stage neoplasms (58). Loss of this gene could be investigated as a potential marker of malignancy in parathyroid tumors and may yield a useful new indicator for the histopathological diagnosis of parathyroid carcinomas. Two genes associated with amyloid protein-dependent signaling perturbations in Alzheimer's disease (AD) may play an important role in Def-Ts and could reveal an intriguing connection linking amyloidosis in aging individuals to disruptions in parathyroid function. MAPK8IP1, which opposes MAPK8-mediated signal transduction, is downregulated in Def-Ts and has been found to colocalize with amyloid deposits in AD neurofibrillary tangles (33). GABBR1, upregulated in Def-Ts, can be biochemically activated by amyloid-derived peptides (59); our group has recently shown that GABBR1 can oppose CaSR signaling in parathyroid tissue by forming CaSR:GABBR1 heteromers that displace calcium-responsive CaSR:CaSR homomers (34). These observations suggest that the reduced expression of MAPK8IP1 and increased levels of GABBR1 in vitamin D-deficient patients could render them more susceptible to increased amyloid protein levels, with enhanced amyloid-initiated aggregation and inactivation of MAPK8IP1 and increased opposition to CaSR signaling through amyloid-liganded GABBR1 activation both contributing to PTH hypersecretion. Interestingly, Rep-Ts also exhibit enrichment for genes associated with beta-amyloid signaling, suggesting that amyloid peptides may exert effects on both classes of parathyroid tumors. Future studies defining the role of amyloid peptides in influencing calcium sensing and PTH secretion in the parathyroid gland will provide a clearer understanding of this previously unrecognized relationship.

Collectively, the parathyroid adenomas in our overall PHPT cohort express elevated levels of genes involved in tissue remodeling, cellular structure, and tumor microenvironment interactions when compared to normal parathyroid tissue. Consistent with the low mitotic index of parathyroid adenomas, the tumors do not share a dominant proliferative signature, but as a group, they appear to mobilize genes that promote morphological processes such as the epithelial–mesenchymal transition (EMT). The enhanced activity of genes in these pathways could enable features common to all parathyroid adenomas such as higher cell density and other tumor-specific structural changes including disruption of stromal boundaries, epithelial cell polarity, and basement membrane attachment. IGFBP5, a gene downregulated in both Def-Ts and Rep-Ts, is an important stimulator of osteogenic differentiation (50). Because microRNA-mediated silencing of IGFBP5 has been shown to promote osteoporosis (49), it is possible that attenuated expression of this gene in parathyroid adenomas could contribute to bone mineral density loss in PHPT in addition to the direct osteoclastic effects of PTH.

Transcriptomic studies of parathyroid tumors to date have largely employed candidate gene analysis or aggregate evaluation of bulk tumor input. As our primary focus was comparing cellular subsets within Def-Ts and Rep-Ts, the principal gene expression differences that we identified are reflective of vitamin D status in age- and sex-matched PHPT patients. Because our current study utilized a spatially indexed approach for whole transcriptome profiling of specific cellular subsets within parathyroid tumors and normal tissue, the results reported here are not directly comparable to previously published work. Nonetheless, certain informative commonalities and distinctions are apparent. Consistent with earlier studies (60, 61), we found that PTH transcript abundance was not elevated in parathyroid adenomas relative to normal tissue, suggesting that the hypersecretory behavior of these tumors is not dependent upon increased PTH gene expression. While we did not observe significantly reduced PTH mRNA levels in parathyroid tumor cells compared to normal tissue, our experimental design utilized a cross-sectional panel of independent normal donor glands as a reference standard rather than tumor-adjacent histologically normal parathyroid cells that may be influenced by the adjoining tumor (60) or parathyroid glands obtained from thyroid carcinoma patients undergoing thyroidectomy (61). Variability between these respective control groups could contribute to the detection of alternative sets of differentially expressed genes when compared to parathyroid adenomas.

Using a fold change threshold of ≥2 and a false discovery cutoff of <0.001, Chai et al (61) identified 247 DEGs (45 up-regulated, 202 down-regulated) with enrichment in KEGG pathways associated with protein processing in the ER, protein export, RNA transport, glycosylphosphotidylinositol-anchor biosynthesis, and pyrimidine metabolism. In our study, we utilized a similar fold change threshold but employed a much more stringent false discovery criterion of 1 × 10^−6^ in order to limit potentially artifactual differences that could arise from closely related interrogative comparisons (i.e. chief cells and oxyphil cells from a single tumor, or PHPT tumors of different vitamin D status, instead of tumor vs normal comparisons where a wider degree of divergence would be anticipated). This increased stringency, coupled with our directed comparisons between the Rep-T and Def-T subsets of PHPT adenomas, allowed us to identify vitamin D-correlated signatures that may not have emerged in aggregate tumor versus normal tissue analysis. Nonetheless, our findings are consistent with Chai et al. in revealing that parathyroid tumors in general do not exhibit a predominant proliferative gene expression profile. These results support the concept that the PHPT disease process is driven more by parathyroid tumor changes in metabolic behavior, alterations in the relationship with the extracellular environment, and protein processing in parathyroid adenomas rather than primarily by mitotically activating oncogenic events. Intriguingly, tumor-specific alterations in the beta amyloid signaling pathway were identified in the Chai et al. report as well as the current study. Further investigation of this novel relationship could yield important new insights into the potential mechanistic function of the beta-amyloid peptide in PHPT.

There are several important limitations to the current study that will warrant further investigation. The cohort of vitamin D-deficient patients is small, since preoperative vitamin D deficiency is frequently restored by supplementation prior to surgery. Future studies of tumors from PHPT patients who initially presented with vitamin D deficiency at the time of diagnosis but were repleted prior to parathyroidectomy could reveal whether the Def-T profile can be dynamically switched by vitamin D therapy. It could prove useful to search for additional factors associated with vitamin D deficiency in PHPT patients to determine whether there are unrecognized variables or comorbidities contributing to their low vitamin D status and clinical presentation. Inclusion of the larger cohort of PHPT patients presenting with vitamin D insufficiency (20 ng/ml < 25OHD < 30 ng/ml) could provide a transitional state for identifying dose-dependent dynamic shifts in gene expression as confirmation of the vitamin D dependency of differentially expressed candidate genes or gene pathways identified in the current study. In future work, we will test the proposed vitamin D-dependent stratification of gene expression patterns in parathyroid cells by incorporating tumors from vitamin D-insufficient patients into our profiling studies for comparison to the vitamin D-deficient and vitamin D-replete groups. Our study drew upon a preexisting registry of female PHPT patients accrued through an ongoing project at our institution. While this experimental design allowed us to remove sex as a factor in our comparisons, future studies evaluating sex as a contributing variable could provide new key insights into the biology of PHPT. Specifically, it will be highly informative to determine whether the increased incidence of PHPT among older women is primarily a consequence of demographic factors or, alternatively, is indicative of important underlying biological differences, including the metabolism and actions of vitamin D in parathyroid tissue in females.

In summary, our study demonstrates that vitamin D deficiency is associated with cellular and transcriptomic changes in parathyroid tissue that could contribute to tumor development in PHPT. Parathyroid adenomas from PHPT patients with preoperative vitamin D deficiency are molecularly distinct from tumors from PHPT patients who are vitamin D replete. Gene set enrichment comparative analysis of Def-Ts and Rep-Ts suggests the possibility of alternative etiologies for these tumors and supports the notion that oxyphil cells in either context are lineal descendants of chief cells. The differential expression in parathyroid tumors of genes associated with beta-amyloid signaling reveals a potential connection between the increased amyloid burden in aging adults (62, 63) and the heightened incidence of PHPT among older individuals (64). Collectively, these findings suggest that PHPT in vitamin D-deficient patients may be a distinct subset of the disease with an alternative etiology, tumor composition, and cellular behavior. Further experiments assessing the reversibility of the tumor phenotype associated with vitamin D deficiency are warranted to determine whether vitamin D supplementation could potentially mitigate the clinical phenotype in PHPT patients presenting with low vitamin D status.

## Materials and methods

### Human parathyroid tissue collection

#### Normal donor parathyroid tissue

Normal human parathyroid tissue was obtained through our institution's solid organ transplant service from an unselected sequential series of eucalcemic donors, using a fully authorized tissue procurement protocol for the recovery of viable, intact parathyroid glands. The vitamin D levels of the donors were not determined. Dissected glands were immediately fixed in 4% paraformaldehyde (PFA), embedded, and sectioned as previously described (65).

#### Parathyroid adenoma collection

Parathyroid adenoma specimens were obtained under an IRB-approved protocol (IRB protocol number 19-27072) from patients undergoing surgery for primary hyperparathyroidism at our high-volume endocrine surgery center. Clinical, demographic, and pathological patient data were collected from the medical record and anonymized by the study clinical research coordinator in compliance with IRB requirements. The tumor samples were fixed, embedded, and sectioned using standard methods (65). Briefly, parathyroid tissue was fixed in 4% paraformaldehyde (PFA) in 0.1 M PBS (pH 7.6) overnight at room temperature. After fixation, the tissue was rinsed with ddH2O and the PFA was replaced with 70% ethanol for storage. The tissue was embedded in paraffin and 5-micron sections were prepared for analysis.

### Image analysis for oxyphil quantitation

Immunofluorescence images of complete tissue sections from each specimen were exported as single-channel TIFF files from the GeoMx Digital Spatial Profiler instrument (NanoString Technologies) and analyzed using ImageJ. The total cell number was quantitated from the nuclear stain SYTO13 channel using the Analyze Particles module of ImageJ. Oxyphils were quantitated from the anti-TOMM20-AlexaFluor594 channel using the 3D Object Counter module of ImageJ. Cell counts were limited to CaSR-positive cells (marked by the anti-CaSR-AlexaFluor647 channel) in each section to exclude vascular elements and other nonparathyroid components. TOMM20 was detected using an AlexaFluor594-conjugated mouse monoclonal antibody (catalog number sc-17764, Santa Cruz) at a concentration of 2 μg/ml. CaSR was detected using an AlexaFluor647-conjugated mouse monoclonal antibody, clone 3H8E9 (34) at a concentration of 5 μg/ml.

### Digital spatial profiling with the GeoMx Human Whole Transcriptome Atlas

Five μm-thick FFPE sections were prepared from normal donor human parathyroid glands or from human parathyroid adenomas resected from patients undergoing parathyroidectomy for primary hyperparathyroidism. The sections were then processed using the GeoMx DS-NGS RNA FFPE slide prep protocol (NanoString Technologies). The slides were first deparaffinized and subjected to heat-inducible antigen retrieval procedures (15 min at 100°C with 1× Tris-EDTA buffer pH 9) and proteinase K digestion (1 μg/ml, 15 min at 37°C). The treated slides were hybridized to the Human Whole Transcriptome Atlas probe set (1 : 12.5 dilution, 16 h at 37°C) and slides were washed twice in fresh 2× saline sodium citrate buffer (SSC). Prior to imaging on the GeoMx Digital Spatial Profiler (DSP) instrument, parathyroid tissue morphology was visualized using fluorescent-labeled antibodies (anti-CaSR and anti-Tomm20) and nuclei were visualized with 500 nM Syto13, a fluorescent DNA stain. Entire slides were imaged at 20× magnification, and 8 to 16 regions of interest (ROI) were selected per sample. ROIs were chosen based on morphology markers (CaSR+/Tomm20+/Syto13+ and CaSR+/Tomm20-/Syto13+ for chief cell-enriched and oxyphil-enriched compartment, respectively). CaSR and TOMM20 positive cells were defined as those with immunofluorescence signal intensity in the top 40% of the signal range for each section stained with anti-CaSR-AF647 or anti-TOMM20-AF594, respectively. Negative cells for each marker were defined as those with immunofluorescence intensity in the bottom 40% of the signal range for each section. The GeoMx instrument was then exposed ROIs to 385 nm UV light, releasing the indexing oligos and collecting them with a microcapillary. Indexing oligos were then deposited in a 96-well plate for subsequent processing. The indexing oligos were dried down overnight and resuspended in 10 μl of diethyl pyrocarbonate (DEPC)-treated water.

Sequencing libraries were generated by PCR from the photo-released indexing oligos and ROI-specific Illumina adapter sequences, and unique i5 and i7 sample indices were added. Each polymerase chain reaction (PCR) used 4 μl of indexing oligos, 4 μl of indexing primer mix, and 2 μl of NanoString 5X PCR Master Mix. Thermocycling conditions were 37°C for 30 min, 50°C for 10 min, and 95°C for 3 min; 18 cycles of 95°C for 15 s, 65°C for 1 min, and 68°C for 30 s; and 68°C for 5 min. PCR reactions were pooled and purified twice using AMPure XP beads (Beckman Coulter, A63881) according to manufacturer's protocol. Pooled libraries were sequenced at 2 × 27 base pairs and with the dual-indexing workflow on an Illumina NextSeq500 sequencer.

### Analysis of human GeoMx DSP data

Gene expression counts were determined using the GeoMx Human Whole Transcriptome Atlas-Human RNA for Illumina Systems (GMX-RNA-NGS-HuWTA-4) RNA probe set. This panel profiles the whole transcriptome by targeting 18,000+ unique transcripts from human protein encoding genes plus ERCC negative controls. The panel excludes uninformative high-abundance RNAs such as ribosomal subunits and includes RNA probes designed for Illumina NGS readout with the Seq Code library prep.

Raw Illumina counts were Q3 normalized using the GeoMx software and standardized QC threshold settings as recommended by the manufacturer. The data were then log2 transformed prior to downstream analysis. Principal component analysis was computed through the *irlba* package in R, using the top 1,000 most differentially expressed genes (DEGs) out of 12,762 unique transcripts detected. T-distributed stochastic neighbor embedding (t-SNE) calculations were performed using the *Rtsne* R package, reducing the top 1,000 DEGs to 50 PCA dimensions before computing the t-SNE embedding. The perplexity was heuristically set to 25% of the sample size. Uniform Manifold Approximation and Projection (UMAP) was computed using the *uwot* package in R, with the same input and heuristic settings. Heat maps were generated using the ComplexHeatmap R/Bioconductor package on scaled log-expression values using Euclidean distance and Ward linkage. The standard deviation was used to rank the genes, with the top 150 genes with the greatest degree of differential expression (largest standard deviation between groups) incorporated into the heatmap.

Statistical testing of differential gene set enrichment was performed using Fisher's exact test (fGSEA), Camera, and GSVA/limma (66). The maximum *q*-value of the three methods was taken as the aggregate *q*-value, which corresponds to taking the intersection of significant genes from all three tests. Gene sets polled were from public databases including Gene Ontology (67, 68), the Kyoto Encyclopedia of Genes and Genomes (KEGG) (69), and MSigDB (70). Enrichment scores were calculated using the GSEA (v14) algorithm (https://www.gsea-msigdb.org/gsea/index.jsp) as previously described (27). Differential expression analysis was performed by comparing groups (tumors from vitamin D-deficient vs vitamin D-replete patients; chief vs oxyphil cells; and normal tissue vs all tumors), with the false discovery rate (FDR) threshold set to 1 × 10^−6^ and the log_2_(fold change) threshold set to ±1.

R-based analysis was performed in the Omics Playground platform (66), implemented in R using the open-source Shiny Server web application framework. The source code for the platform was cloned from a publicly available GitHub repository (https://github.com/bigomics/omicsplayground.git).

### GeoMx DSP protein nCounter quantitation

Five µm-thick FFPE sections were prepared using the GeoMx DSP Protein slide prep protocol (NanoString Technologies). Briefly, the slides were first deparaffinized and subjected to standard heat-inducible antigen retrieval procedures (15 min at ∼95°C in 1× pH6 citrate buffer in a pressure cooker). The slides were then coincubated with fluorescent-conjugated morphology marker antibodies (as described above in the GeoMx DSP transcriptome methods), together with photocleavable oligonucleotide-labeled primary antibodies (profiling antibodies, see below), followed by incubation with 500 nM Syto13 nuclear stain. The stained slides were then loaded into the GeoMx DSP instrument and were scanned at 20× magnification to produce a digital fluorescence image of the entirety of the tissue sections on each slide. Circular regions of interest (660 µM^2^) were selected to capture roughly equivalent numbers of chief and oxyphil cells as described above. To obtain cell type-specific protein measurements, we utilized generated molecularly defined compartments within each ROI using the TOMM20 and CaSR morphology markers described above. Chief cells and oxyphil cells were marked and oligonucleotide tags corresponding to bound antibodies within each cell compartment were released by UV (385 nm) photocleavage. The released oligonucleotides were recovered and dispensed into 96-well plates. The indexing oligonucleotides were dried down and resuspended in 7 μl of diethyl pyrocarbonate (DEPC)-treated water, hybridized to 4-color, 6-spot optical barcodes, and digitally counted using the nCounter system (NanoString Technologies). GeoMx software was used to normalize the digital counts using internal spike-in controls (ERCCs) and a housekeeping gene panel as previously described (71).

The profiling antibodies were from a custom GPCR module that includes internal GeoMX DSP controls and antibodies to detect CaSR (MAb 1C12D7), metabotropic gamma-aminobutyric acid (GABA) receptors GABBR1 (Abcam, ab264069, RabMAb EPR22954-47), and GABBR2 (Abcam, ab230136, RabMAb EP2411).

## Supplementary Material

pgad073_Supplementary_Data

## Data Availability

The data supporting this work include private health information from the medical records of the study participants. As mandated under the IRB protocol authorizing the study, these data cannot be released publicly. Supporting data in the fully anonymized form will be made available upon request from the corresponding author after publication.

## References

[1] Boudou P, Ibrahim F, Cormier C, Sarfati E, Souberbielle JC. 2006. A very high incidence of low 25 hydroxy-vitamin D serum concentration in a French population of patients with primary hyperparathyroidism. J Endocrinol Invest. 29:511–515. 16840828 10.1007/BF03344140

[2] Walker MD, Bilezikian JP. 2017. Vitamin D and primary hyperparathyroidism: more insights into a complex relationship. Endocrine. 55:3–5. 27858283 10.1007/s12020-016-1169-1 PMC5226876

[3] Moosgaard B, et al 2005. Vitamin D status, seasonal variations, parathyroid adenoma weight and bone mineral density in primary hyperparathyroidism. Clin Endocrinol (Oxf). 63:506–513. 16268801 10.1111/j.1365-2265.2005.02371.x

[4] Moosgaard B, et al 2008. Vitamin D metabolites and skeletal consequences in primary hyperparathyroidism. Clin Endocrinol (Oxf). 68:707–715. 17980013 10.1111/j.1365-2265.2007.03109.x

[5] Harkness L, Cromer B. 2005. Low levels of 25-hydroxy vitamin D are associated with elevated parathyroid hormone in healthy adolescent females. Osteoporos Int. 16:109–113. 15175848 10.1007/s00198-004-1656-8

[6] Raposo L, Martins S, Ferreira D, Guimaraes JT, Santos AC. 2017. Vitamin D, parathyroid hormone and metabolic syndrome - the PORMETS study. BMC Endocr Disord. 17:71. 29149839 10.1186/s12902-017-0221-3 PMC5693479

[7] Clements MR, et al 1992. The role of 1,25-dihydroxyvitamin D in the mechanism of acquired vitamin D deficiency. Clin Endocrinol (Oxf). 37:17–27. 1424188 10.1111/j.1365-2265.1992.tb02278.x

[8] Silver J, Russell J, Sherwood LM. 1985. Regulation by vitamin D metabolites of messenger ribonucleic acid for preproparathyroid hormone in isolated bovine parathyroid cells. Proc Natl Acad Sci U S A. 82:4270–4273. 3858880 10.1073/pnas.82.12.4270 PMC397979

[9] Kremer R, Bolivar I, Goltzman D, Hendy GN. 1989. Influence of calcium and 1,25-dihydroxycholecalciferol on proliferation and proto-oncogene expression in primary cultures of bovine parathyroid cells. Endocrinology. 125:935–941. 2502380 10.1210/endo-125-2-935

[10] Meir T, et al 2009. Deletion of the vitamin D receptor specifically in the parathyroid demonstrates a limited role for the receptor in parathyroid physiology. Am J Physiol Renal Physiol. 297:F1192–F1198. 19692484 10.1152/ajprenal.00360.2009

[11] Ritter CS, Haughey BH, Miller B, Brown AJ. 2012. Differential gene expression by oxyphil and chief cells of human parathyroid glands. J Clin Endocrinol Metab. 97:E1499–E1505. 22585091 10.1210/jc.2011-3366 PMC3591682

[12] Li S, et al 2018. Comparative proteomic analysis of chief and oxyphil cell nodules in refractory uremic hyperparathyroidism by iTRAQ coupled LC-MS/MS. J Proteomics. 179:42–52. 29526777 10.1016/j.jprot.2018.02.029

[13] Christie AC . 1967. The parathyroid oxyphil cells. J Clin Pathol. 20:591–602. 4880406 10.1136/jcp.20.4.591 PMC473518

[14] Shi Y, Hogue J, Dixit D, Koh J, Olson JA Jr. 2014. Functional and genetic studies of isolated cells from parathyroid tumors reveal the complex pathogenesis of parathyroid neoplasia. Proc Natl Acad Sci U S A. 111:3092–3097. 24510902 10.1073/pnas.1319742111 PMC3939880

[15] Koh J, et al 2018. Transcriptional profiling reveals distinct classes of parathyroid tumors in PHPT. Endocr Relat Cancer. 25:407–420. 29475894 10.1530/ERC-17-0470 PMC5826637

[16] Mao J, et al 2022. Integrated transcriptomic and proteomic analyses for the characterization of parathyroid oxyphil cells in uremic patients. Amino Acids. 54:749–763. 35348903 10.1007/s00726-022-03126-8

[17] Bedetti CD, Dekker A, Watson CG. 1984. Functioning oxyphil cell adenoma of the parathyroid gland: a clinicopathologic study of ten patients with hyperparathyroidism. Hum Pathol. 15:1121–1126. 6389314 10.1016/s0046-8177(84)80306-8

[18] Erbil Y, et al 2008. The positive effect of adenoma weight and oxyphil cell content on preoperative localization with 99mTc-sestamibi scanning for primary hyperparathyroidism. Am J Surg. 195:34–39. 18082541 10.1016/j.amjsurg.2007.01.040

[19] Fleischer J, Becker C, Hamele-Bena D, Breen TL, Silverberg SJ. 2004. Oxyphil parathyroid adenoma: a malignant presentation of a benign disease. J Clin Endocrinol Metab. 89:5948–5951. 15579742 10.1210/jc.2004-1597

[20] Howson P, et al 2015. Oxyphil cell parathyroid adenomas causing primary hyperparathyroidism: a clinico-pathological correlation. Endocr Pathol. 26:250–254. 26091632 10.1007/s12022-015-9378-3

[21] Metgudmath RB, Metgudmath VV, Malur PR, Das AT, Metgudmath AR. 2014. Functioning oxyphil parathyroid adenoma: a case report. J Clin Diagn Res. 8:QD07-08. 10.7860/JCDR/2014/7724.4297 PMC4064877 24959490

[22] Chang W, et al 2020. PTH hypersecretion triggered by a GABA(B1) and Ca(2+)-sensing receptor heterocomplex in hyperparathyroidism. Nat Metab. 2:243–255. 32694772 10.1038/s42255-020-0175-z PMC7377265

[23] Ross AC, et al 2011. The 2011 report on dietary reference intakes for calcium and vitamin D from the Institute of Medicine: what clinicians need to know. J Clin Endocrinol Metab. 96:53–58. 21118827 10.1210/jc.2010-2704 PMC3046611

[24] Walker MD, et al 2015. Vitamin D in primary hyperparathyroidism: effects on clinical, biochemical, and densitometric presentation. J Clin Endocrinol Metab. 100:3443–3451. 26079779 10.1210/jc.2015-2022 PMC4570160

[25] Costa-Guda J, Tokura T, Roth SI, Arnold A. 2007. Mitochondrial DNA mutations in oxyphilic and chief cell parathyroid adenomas. BMC Endocr Disord. 7:8. 17916247 10.1186/1472-6823-7-8 PMC2099428

[26] Bhattacharya A, et al 2021. An approach for normalization and quality control for NanoString RNA expression data. Brief Bioinform. 22:bbaa163. 32789507 10.1093/bib/bbaa163 PMC8138885

[27] Mootha VK, et al 2003. PGC-1alpha-responsive genes involved in oxidative phosphorylation are coordinately downregulated in human diabetes. Nat Genet. 34:267–273. 12808457 10.1038/ng1180

[28] Liu S, et al 2021. Three differential expression analysis methods for RNA sequencing: limma, EdgeR, DESeq2. J Vis Exp. (175). 10.3791/62528 34605806

[29] Maza E . 2016. In papyro comparison of TMM (edgeR), RLE (DESeq2), and MRN normalization methods for a simple two-conditions-without-replicates RNA-Seq experimental design. Front Genet. 7:164. 27695478 10.3389/fgene.2016.00164 PMC5025571

[30] Varet H, Brillet-Gueguen L, Coppee JY, Dillies MA. 2016. SARTools: a DESeq2- and EdgeR-based R pipeline for comprehensive differential analysis of RNA-Seq data. PLoS One. 11:e0157022. 27280887 10.1371/journal.pone.0157022 PMC4900645

[31] Son HJ, Choi EJ, Yoo NJ, Lee SH. 2020. Mutation and expression of a candidate tumor suppressor gene EPB41L3 in gastric and colorectal cancers. Pathol Oncol Res. 26:2003–2005. 31828581 10.1007/s12253-019-00787-x

[32] Giuliano AR, et al 2020. Methylation of HPV 16 and EPB41L3 in oral gargles: associations with oropharyngeal cancer detection and tumor characteristics. Int J Cancer. 146:1018–1030. 31304592 10.1002/ijc.32570 PMC7787351

[33] Beeler N, Riederer BM, Waeber G, Abderrahmani A. 2009. Role of the JNK-interacting protein 1/islet brain 1 in cell degeneration in Alzheimer disease and diabetes. Brain Res Bull. 80:274–281. 19616077 10.1016/j.brainresbull.2009.07.006

[34] Chang W, et al 2020. PTH hypersecretion triggered by a GABAB1 and Ca2+ -sensing receptor heterocomplex in hyperparathyroidism. Nat Metab. 2:243–255. 32694772 10.1038/s42255-020-0175-z PMC7377265

[35] Hofle J, et al 2022. Engagement of TRAIL triggers degranulation and IFNγ production in human natural killer cells. EMBO Rep. 23:e54133. 10.15252/embr.202154133 PMC9346491 35758160

[36] Airiau K, et al 2021. TRAIL triggers CRAC-dependent calcium influx and apoptosis through the recruitment of autophagy proteins to death-inducing signaling complex. Cells. 11:57. 35011619 10.3390/cells11010057 PMC8750441

[37] Ghani MJ . 2022. SGK1, autophagy and cancer: an overview. Mol Biol Rep. 49:675–685. 34669124 10.1007/s11033-021-06836-6

[38] Kim E, Ahn H, Kim MG, Lee H, Kim S. 2017. The expanding significance of inositol polyphosphate multikinase as a signaling hub. Mol Cells. 40:315–321. 28554203 10.14348/molcells.2017.0066 PMC5463039

[39] Hsu CM, et al 2022. Down-regulation of AMPD3 is associated with poor survival in head and neck squamous cell carcinoma. In Vivo. 36:704–712. 35241525 10.21873/invivo.12756 PMC8931920

[40] Groulx JF, et al 2011. Collagen VI is a basement membrane component that regulates epithelial cell-fibronectin interactions. Matrix Biol. 30:195–206. 21406227 10.1016/j.matbio.2011.03.002

[41] Saito J, Ishikawa Y, Yokoyama U. 2020. Role of tissue-type plasminogen activator in remodeling of the ductus arteriosus. Circ Rep. 2:211–217. 33693232 10.1253/circrep.CR-20-0015 PMC7921361

[42] Chen B, et al 2016. XB130 is overexpressed in prostate cancer and involved in cell growth and invasion. Oncotarget. 7:59377–59387. 27509056 10.18632/oncotarget.11074 PMC5312318

[43] Shen J, et al 2017. XB130 enhances invasion and migration of human colorectal cancer cells by promoting epithelialmesenchymal transition. Mol Med Rep. 16:5592–5598. 28849225 10.3892/mmr.2017.7279

[44] Poosekeaw P, et al 2021. Adaptor protein XB130 regulates the aggressiveness of cholangiocarcinoma. PLoS One. 16:e0259075. 34780466 10.1371/journal.pone.0259075 PMC8592414

[45] Kalantari E, et al 2022. Significant co-expression of putative cancer stem cell markers, EpCAM and CD166, correlates with tumor stage and invasive behavior in colorectal cancer. World J Surg Oncol. 20:15. 35016698 10.1186/s12957-021-02469-y PMC8751119

[46] Brinkhof B, Zhang B, Cui Z, Ye H, Wang H. 2020. ALCAM (CD166) as a gene expression marker for human mesenchymal stromal cell characterisation. Gene. 763S:100031. 34493362 10.1016/j.gene.2020.100031

[47] Cardenes B, et al 2022. ALCAM/CD166 is involved in the binding and uptake of cancer-derived extracellular vesicles. Int J Mol Sci. 23:5753. 35628559 10.3390/ijms23105753 PMC9143639

[48] Tessema M, et al 2012. Differential epigenetic regulation of TOX subfamily high mobility group box genes in lung and breast cancers. PLoS One. 7:e34850. 22496870 10.1371/journal.pone.0034850 PMC3319602

[49] Zhou JG, et al 2020. MicroRNA-1286 inhibits osteogenic differentiation of mesenchymal stem cells to promote the progression of osteoporosis via regulating FZD4 expression. Eur Rev Med Pharmacol Sci. 24:1–10. 31957812 10.26355/eurrev_202001_19889

[50] Zhang ZM, Min L, Jiang DL, Han ZY, Wang LH. 2021. Insulin-like growth factor binding protein 5: an important regulator of early osteogenic differentiation of hMSCs. Folia Biol (Praha). 67:118–125. 35151245 10.14712/fb2021067030118

[51] Bilezikian JP . 2018. Primary hyperparathyroidism. J Clin Endocrinol Metab. 103:3993–4004. 30060226 10.1210/jc.2018-01225 PMC6182311

[52] Viccica G, Cetani F, Vignali E, Miccoli M, Marcocci C. 2017. Impact of vitamin D deficiency on the clinical and biochemical phenotype in women with sporadic primary hyperparathyroidism. Endocrine. 55:256–265. 27033542 10.1007/s12020-016-0931-8

[53] Battista C, et al 2017. Vitamin D status in primary hyperparathyroidism: effect of genetic background. Endocrine. 55:266–272. 27154872 10.1007/s12020-016-0974-x

[54] Ghandur-Mnaymneh L, Kimura N. 1984. The parathyroid adenoma. A histopathologic definition with a study of 172 cases of primary hyperparathyroidism. Am J Pathol. 115:70–83. 6711681 PMC1900356

[55] Wieneke JA, Smith A. 2008. Parathyroid adenoma. Head Neck Pathol. 2:305–308. 20614300 10.1007/s12105-008-0088-8 PMC2807581

[56] Arnold A, et al 2002. Molecular pathogenesis of primary hyperparathyroidism. J Bone Miner Res. 17(Suppl 2):N30–N36. 12412775

[57] Zeng R, et al 2018. EPB41L3 is a potential tumor suppressor gene and prognostic indicator in esophageal squamous cell carcinoma. Int J Oncol. 52:1443–1454. 29568917 10.3892/ijo.2018.4316 PMC5873871

[58] Zhu H, et al 2020. Oncogene-induced senescence: from biology to therapy. Mech Ageing Dev. 187:111229. 32171687 10.1016/j.mad.2020.111229

[59] Rice HC, et al 2019. Secreted amyloid-beta precursor protein functions as a GABABR1a ligand to modulate synaptic transmission. Science. 363:eaao4827. 30630900 10.1126/science.aao4827 PMC6366617

[60] Haglund F, et al 2016. Diffuse parathyroid hormone expression in parathyroid tumors argues against important functional tumor subclones. Eur J Endocrinol. 174:583–590. 26865585 10.1530/EJE-15-1062 PMC5081673

[61] Chai YJ, et al 2019. Comparative gene expression profiles in parathyroid adenoma and normal parathyroid tissue. J Clin Med. 8:297. 30832348 10.3390/jcm8030297 PMC6463127

[62] Rodrigue KM, et al 2012. β-Amyloid burden in healthy aging: regional distribution and cognitive consequences. Neurology. 78:387–395. 22302550 10.1212/WNL.0b013e318245d295 PMC3280058

[63] Tasaki M, et al 2021. Age-related amyloidosis outside the brain: a state-of-the-art review. Ageing Res Rev. 70:101388. 34116224 10.1016/j.arr.2021.101388

[64] Minisola S, et al 2022. Epidemiology, pathophysiology, and genetics of primary hyperparathyroidism. J Bone Miner Res. 37:2315–2329. 10.1002/jbmr.4665 36245271 PMC10092691

[65] Kelly YM, et al 2022. Effects of multi-stage procurement on the viability and function of human donor parathyroid glands. J Surg Res. 276:404–415. 35468367 10.1016/j.jss.2022.03.014

[66] Akhmedov M, Martinelli A, Geiger R, Kwee I. 2020. Omics playground: a comprehensive self-service platform for visualization, analytics and exploration of Big Omics Data. NAR Genom Bioinform. 2:lqz019. 33575569 10.1093/nargab/lqz019 PMC7671354

[67] Ashburner M, et al 2000. Gene Ontology: tool for the unification of biology. The Gene Ontology Consortium. Nat Genet. 25:25–29. 10802651 10.1038/75556 PMC3037419

[68] Gene Ontology Consortium . 2021. The Gene Ontology resource: enriching a GOld mine. Nucleic Acids Res. 49:D325–D334. 33290552 10.1093/nar/gkaa1113 PMC7779012

[69] Kanehisa M, Goto S. 2000. KEGG: Kyoto Encyclopedia of Genes and Genomes. Nucleic Acids Res. 28:27–30. 10592173 10.1093/nar/28.1.27 PMC102409

[70] Liberzon A, et al 2015. The Molecular Signatures Database (MSigDB) hallmark gene set collection. Cell Syst. 1:417–425. 26771021 10.1016/j.cels.2015.12.004 PMC4707969

[71] Moutafi MK, et al 2022. Spatially resolved proteomic profiling identifies tumor cell CD44 as a biomarker associated with sensitivity to PD-1 axis blockade in advanced non-small-cell lung cancer. J Immunother Cancer. 10:e004757. 36002182 10.1136/jitc-2022-004757 PMC9413286

