# Correction to: Digital spatial profiling of human parathyroid tumors reveals cellular and molecular alterations linked to vitamin D deficiency

**DOI:** 10.1093/pnasnexus/pgad371

**Published:** 2023-11-15

This is a correction to: Chia-Ling Tu, Wenhan Chang, Julie A Sosa, James Koh, Digital spatial profiling of human parathyroid tumors reveals cellular and molecular alterations linked to vitamin D deficiency, *PNAS Nexus*, Volume 2, Issue 3, March 2023, pgad073, https://doi.org/10.1093/pnasnexus/pgad073

During a retroactive audit conducted by *PNAS Nexus*, it was discovered that this paper was missing a statement acknowledging compliance with the *PNAS Nexus* Human and Animal Participants and Clinical Trials policy:

Our full study protocol was approved by the Human Research Protection Program Institutional Review Board (IRB) at UCSF. Fully informed consent was obtained from all study participants as required by the IRB protocol.

This error has been corrected in the original article.

