# Supplementary material for: Digital spatial profiling of human parathyroid tumors reveals cellular and molecular alterations linked to vitamin D deficiency
Source: PNAS Nexus. 2023 Mar 9;2(3):pgad073. doi: 10.1093/pnasnexus/pgad073 (PMC10042281; doi:10.1093/pnasnexus/pgad073)
Supplement: pgad073_Supplementary_Data [file pgad073_supplementary_data.pdf]

Table S1. Sequencing Saturation by sample

Normal	Deficient	Replete
60.15	57.18	56.22
54.48	64.92	56.83
61.28	55.96	57.73
62.71	55.92	56.61
55.15	66.04	62.37
58.22	60.31	63.94
54.86	56.32	59.44
56.32	59.99	61.81
62.44	62.48	64.48
60.78		62.68
62.48		64.93
60.28		55.42
		59.47
		59.82
		56.63
		62.37
		62.45
		54.28
		60.32
		62.47
		60.31
		62.45
		55.95
		66.03
		56.83
		62.45
		62.7
		58.26
		56.22
		63.93
		54.49
		54.27
		61.28
		59.47

Means not significantly different by ANOVA. (p=0.7943)

Tu et al

Figure S1

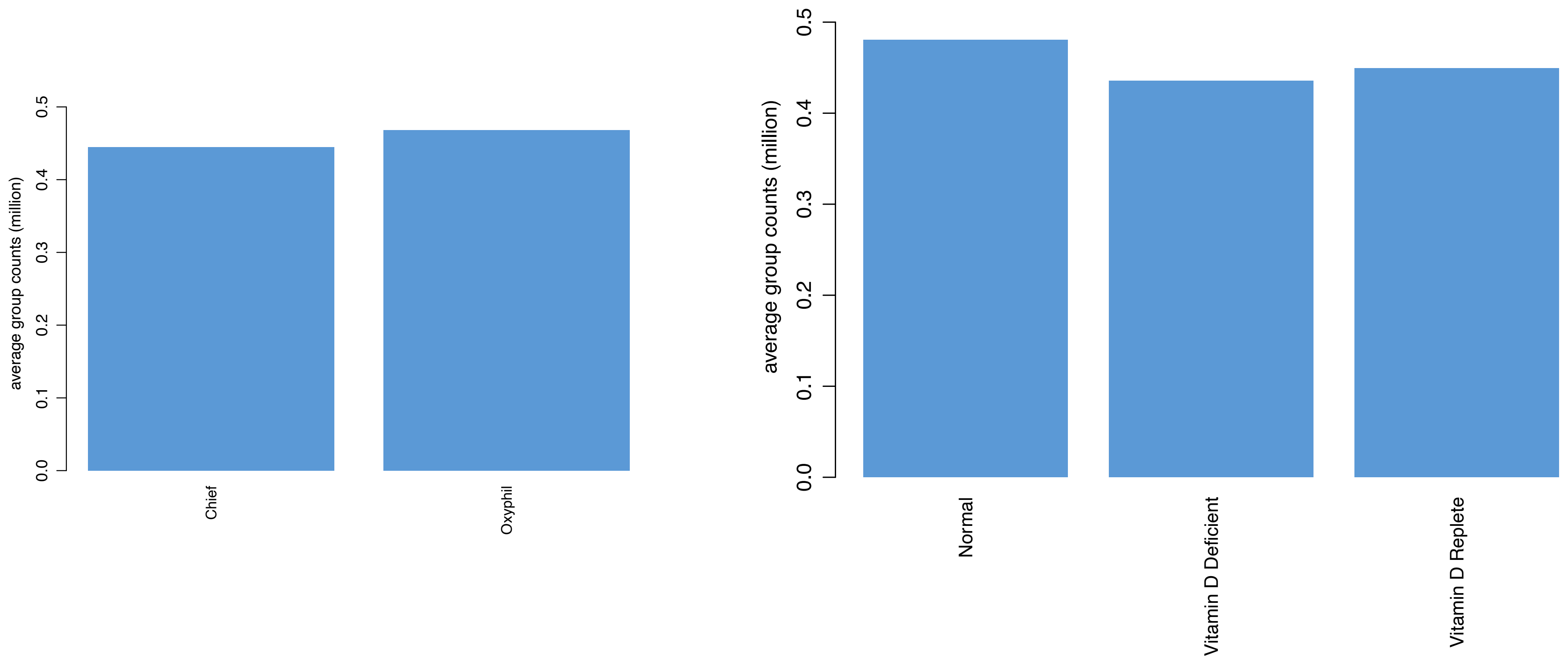

Fig. S1. Average total transcript counts in each cell type (left panel) or in each tissue source (right panel). Counts are expressed in millions.

Tu et al
Figure S1b

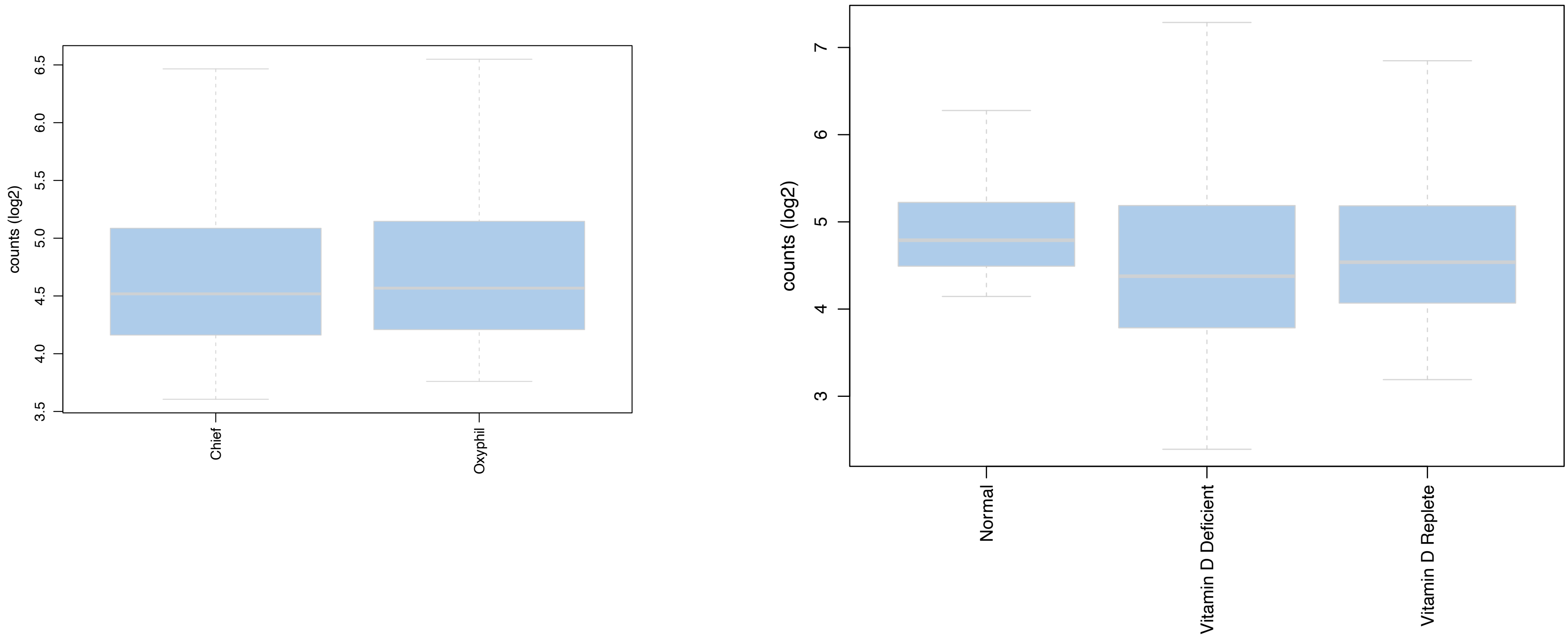

Fig. S1b. Mean counts per transcript (counts distribution) for each cell type (left panel) and for each tissue source (right panel). Gray horizontal line represents the mean; the upper and lower bounds of the blue bars demarcate the 75th and 25th quartile boundaries, respectively. Error bars = SD.

Tu et al
Figure S2

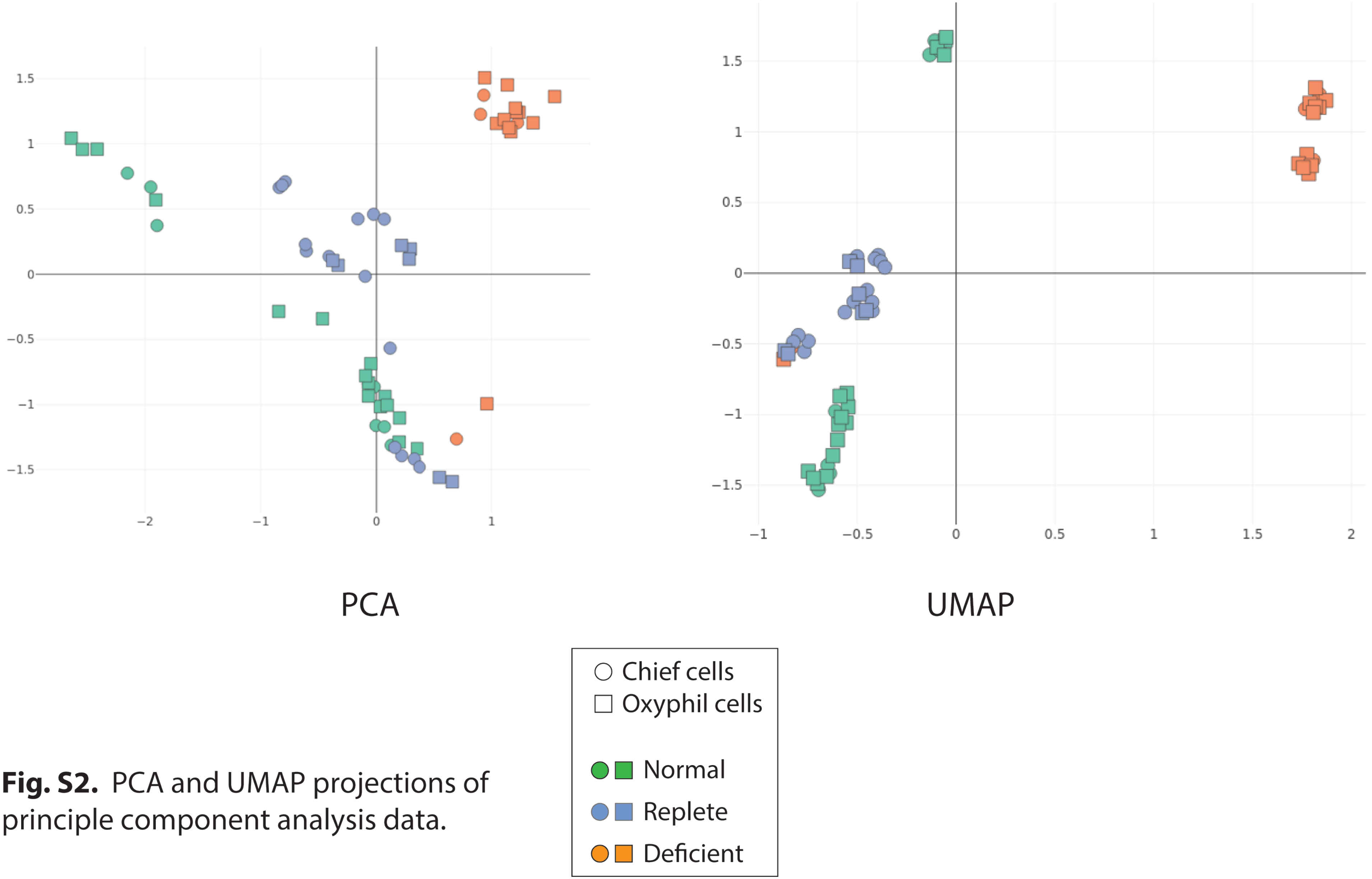

Fig. S2. PCA and UMAP projections of principle component analysis data.
